# Rare Mucinous Tumors of the Alveolar Space: Two Case Reports

**DOI:** 10.1155/crip/6177155

**Published:** 2025-12-11

**Authors:** Calista Sha, Michael Esposito, Paul C. Lee

**Affiliations:** ^1^ Department of Cardiothoracic Surgery, Long Island Jewish Medical Center, New Hyde Park, New York, USA; ^2^ Pathology, Northwell Health Systems, Manhasset, New York, USA

## Abstract

Pulmonary mucinous adenocarcinomas are rare, and the knowledge about them is gleaned from case reports and small case series. To support the growing recognition of these rare tumors, we present two cases: one of a low‐grade mucinous tumor and another of a colloid adenocarcinoma to illuminate the underreported differences between these two mucinous tumors.

## 1. Introduction

Adenocarcinoma is the most common histopathological type of non‐small‐cell lung cancer (NSCLC) [1]. Mucin‐producing adenocarcinomas of the lung are extremely rare, accounting for less than 5% of all lung cancers [2]. They are characterized by an overproduction of mucin, the main component of mucus. Mucus is a protein that help with the function of healthy cells; in mucinous carcinoma, it becomes part of the tumor.

There are two types of mucinous cancers with mucin‐producing properties: mucinous adenocarcinoma, formerly known as bronchioalveolar carcinoma (BAC), or colloid adenocarcinoma [3, 4]. According to the fifth edition of the World Health Organization (WHO) classification of thoracic tumors, mucinous adenocarcinoma has three subcategories: adenocarcinoma in situ, minimally invasive adenocarcinoma, and invasive mucinous adenocarcinoma [5]. Colloid adenocarcinoma is a closely related but distinct type of cancer.

The categorization of mucinous adenocarcinomas has been consistently redefined to be more specific. Recently, there has been a proposal to rename mucinous goblet‐cell rich adenocarcinomas as low‐grade mucinous tumors [6]. In support of the growing efforts to categorize mucinous adenocarcinomas, we present two cases: one of a 71‐year‐old male with a low‐grade mucinous tumor and another of a 70‐year‐old woman with colloid adenocarcinoma. We provide a review of literature, comparing mucinous and colloid adenocarcinomas, and offer our thoughts regarding the reclassification of low‐grade mucinous tumors.

### 1.1. Case 1: Low‐Grade Mucinous Tumor

A 71‐year‐old Russian‐speaking male patient with a history of hypertension, hyperlipidemia, GERD, adenomyomas in the gallbladder, and lung cancer presented to the thoracic surgery clinic for a follow‐up visit. He was a former smoker of 15 pack‐years and had quit nearly a decade ago when he underwent a right middle lobe lobectomy and mediastinal lymph node dissection for pathologically proven Stage IA adenocarcinoma of the lung. Surgical margins were clear of any tumor, and collected lymph nodes were negative for malignancy. He was being annually followed by the thoracic surgery team when his latest routine chest CT revealed a new progressively enlarging 1‐cm semisolid nodule in the right lower lobe. Three additional subcentimeter solid and ground‐glass opacities were considered stable due to unimpressionable changes in size or density on surveillance imaging over the years. The patient reported feeling well and denied symptoms such as shortness of breath, cough, or chest pain. Due to the increasing size of the right lower lobe nodule, a right video‐assisted thoracoscopic surgery (VATS) wedge resection and mediastinal lymph node dissection was done.

**Figure 1:**
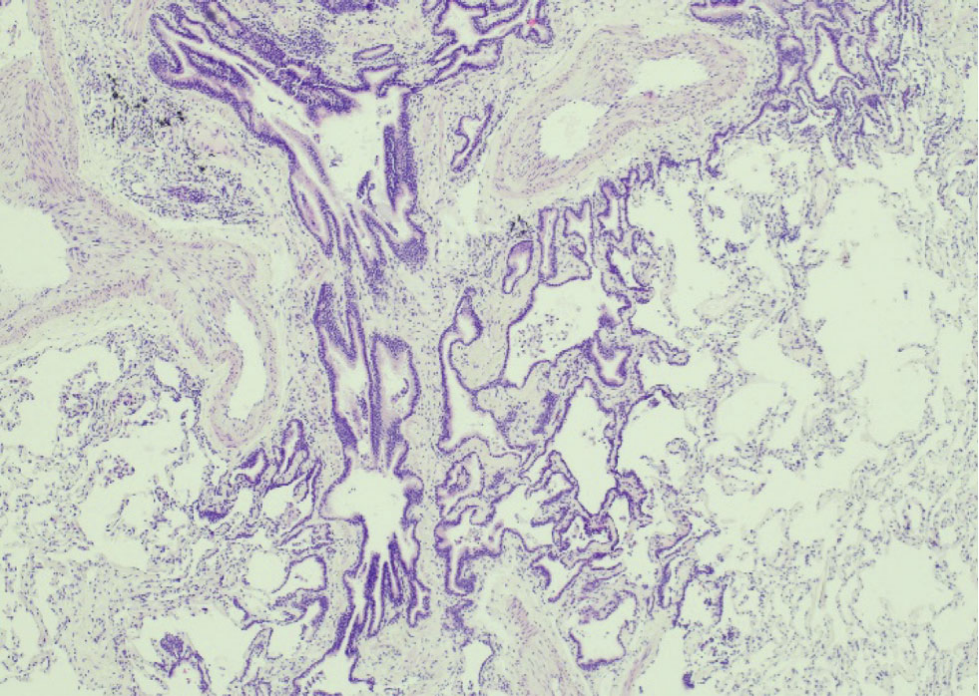
40x H&E peribronchiolar metaplasia.

Lung tissue analysis showed marked peribronchiolar metaplasia (Figure 1) and a 0.9‐cm pluricellular focus of discontinuous, nonatypical colonic‐type glands in an area of fibrosis and mild inflammation (Figure 2). Surgical margins were clear of any tumoral involvement. The glands were negative for TTF‐1 and p40 but strongly positive for CDX2 (Figure 3). The lesion was best classified as a low‐grade mucinous tumor.

Figure 2(a) 40x H&E atypical glands (arrow). (b) H&E 200x infiltrating atypical enteric glands.

**Figure:**
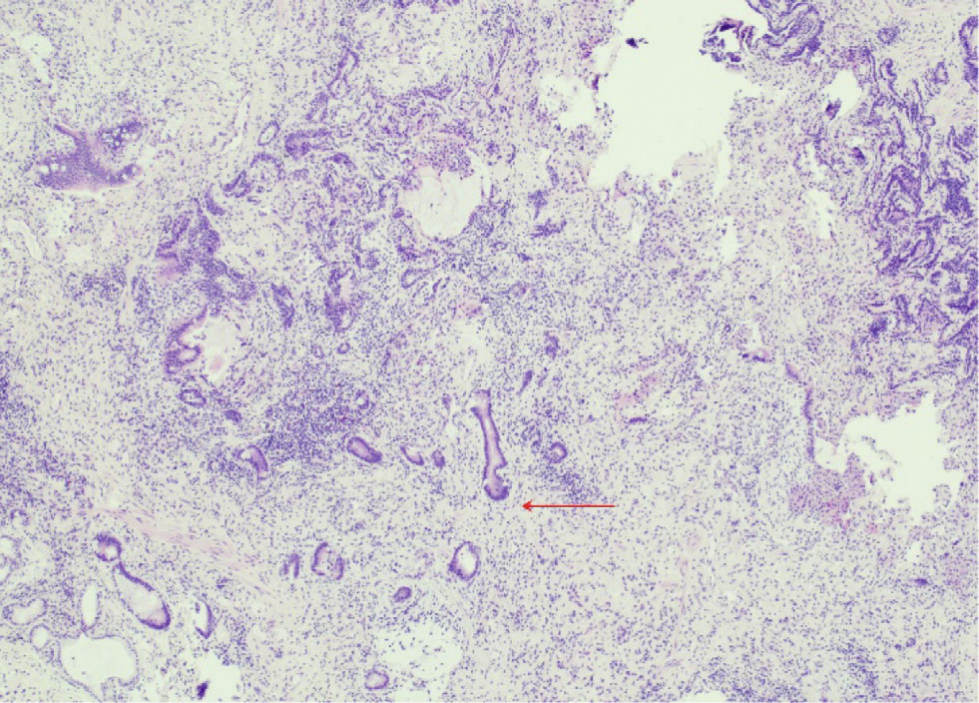
(a)

**Figure:**
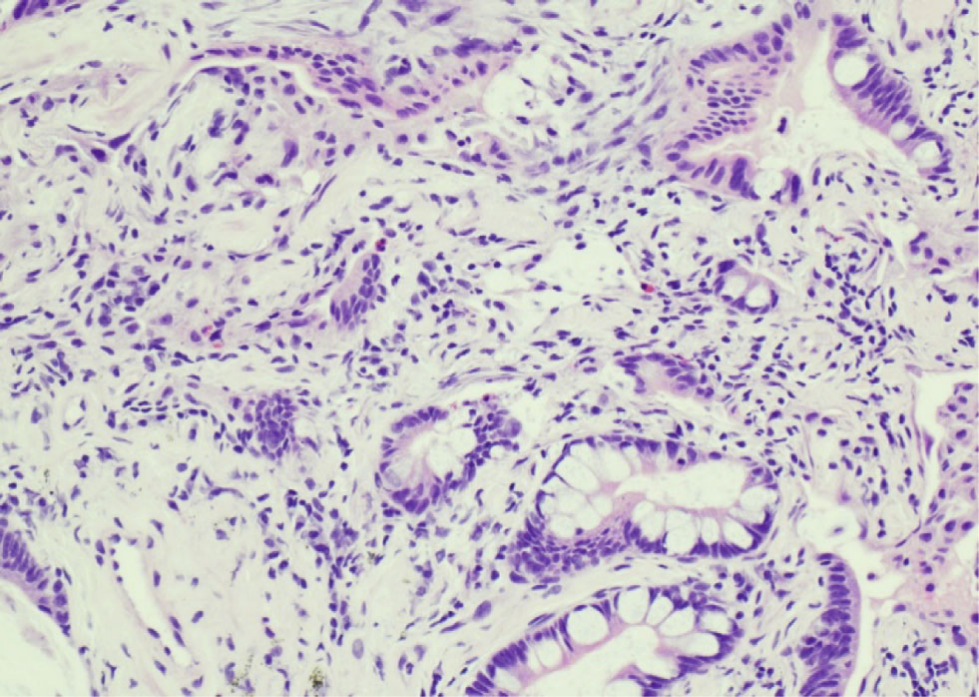
(b)

Figure 3(a) TTF1 100x negative in enteric glands. (b) CDX2 100x positive in enteric glands.

**Figure:**
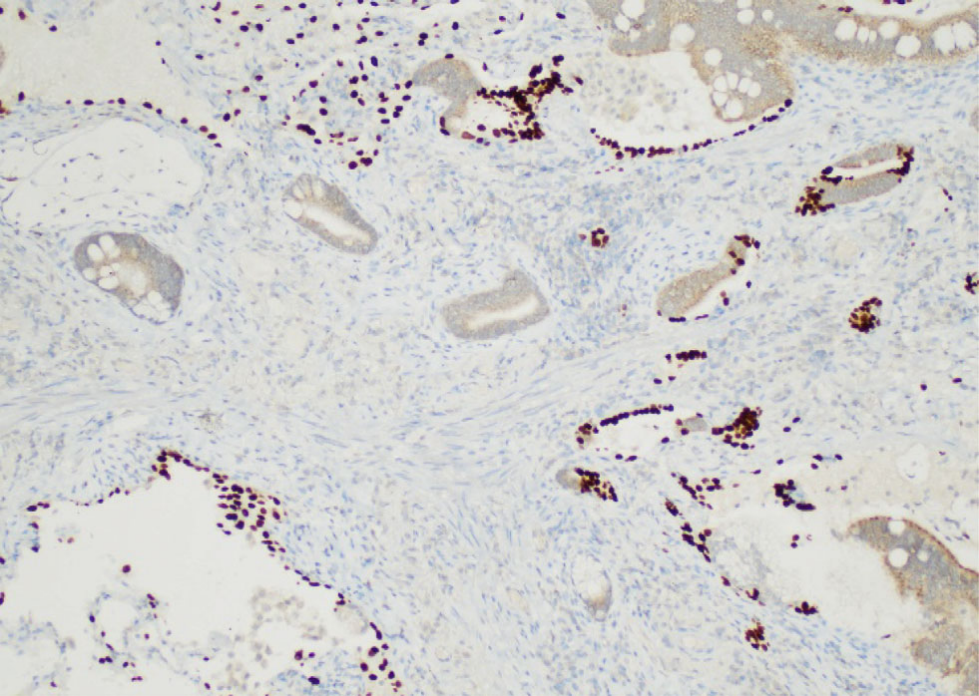
(a)

**Figure:**
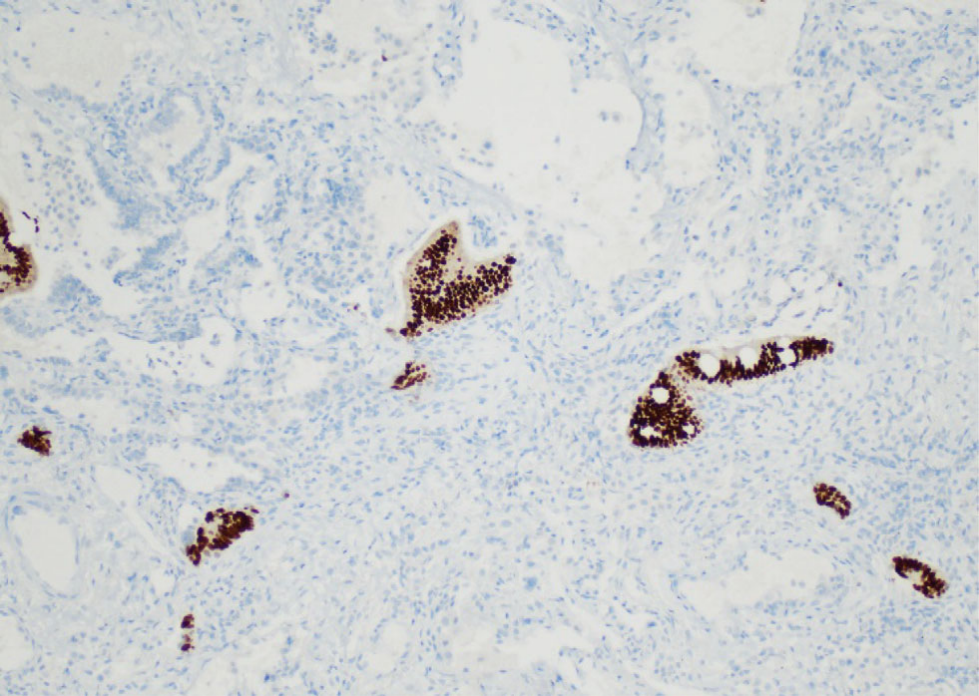
(b)

Then, 6 months postsurgery, the patient is doing well and has no evidence of recurrence.

### 1.2. Case 2: Colloid Adenocarcinoma

A 70‐year‐old woman who was a former smoker with a history of hypertension, hypothyroidism, and breast cancer presented with right‐sided abdominal pain to her primary care physician who ordered a chest x‐ray and incidentally found a left‐sided lung mass.

A chest CT showed a large lobulated circumscribed soft tissue mass 7.6 × 4.2 × 3.1 cm in the left lower lobe, which radiated from the superior margin left hilum towards the posterior lateral pleural margin in the left apex. This abutted and slightly deformed the left interlobar fissure.

A left VATS lobectomy of the left upper lobe with en bloc wedge resection of the posterior left lower lobe was done. Pathology revealed a Stage IIB colloid carcinoma spanning 6.6 cm (Figure 4). All surgical margins were negative for tumoral involvement. Immunostains demonstrated tumor cells were positive for CK7 and CDX2, but negative for CK20, TTF‐1, and GATA‐3. Immunohistochemical stain for PD‐L1 was positive in less than 1% of the neoplastic cells.

Figure 4Mucinous carcinoma colloid variant: (a) 100x and (b) 200x.

**Figure:**
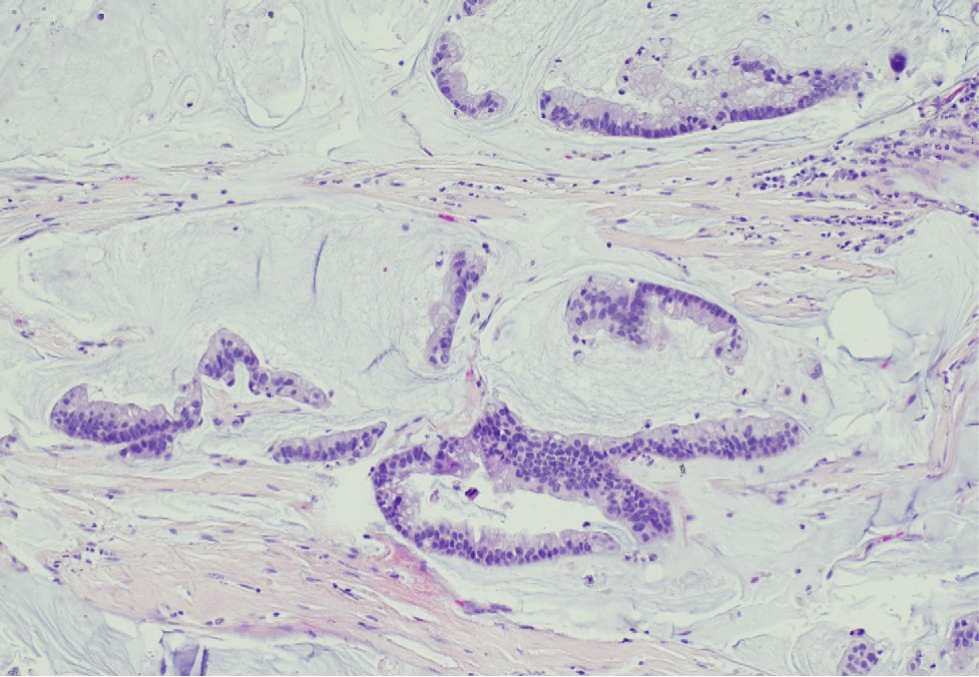
(a)

**Figure:**
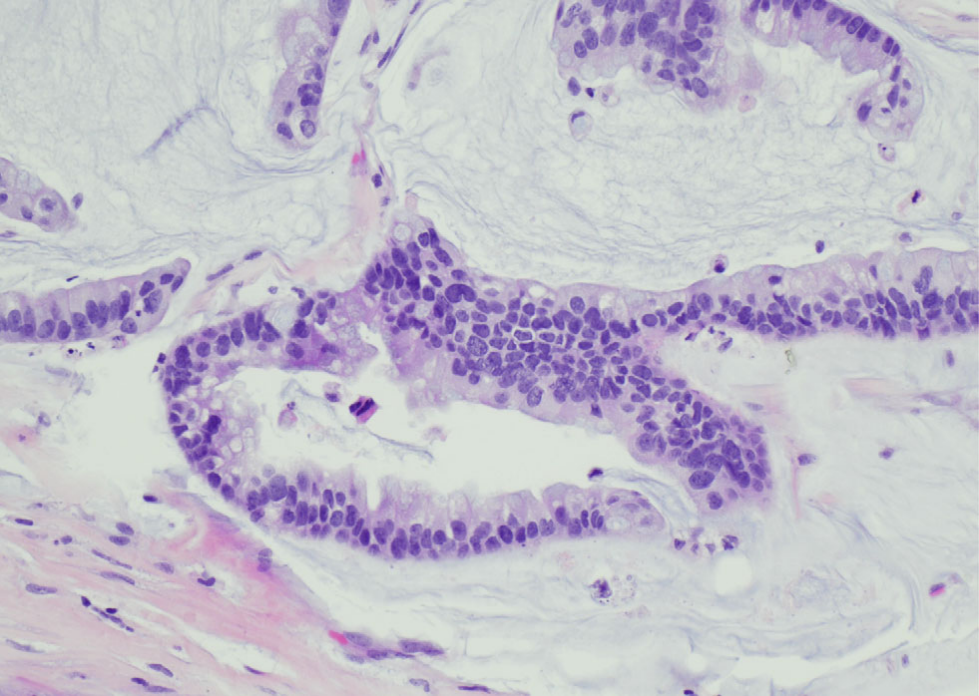
(b)

She completed four cycles of chemotherapy. Then, 2 years postsurgery, she is doing well and has no evidence of recurrence.

## 2. Discussion

Pulmonary adenocarcinomas are the most common type of NSCLC, composing about 40% of all lung cancers. Mucin‐producing adenocarcinomas are the rarest subtypes of lung adenocarcinoma and are defined by the presence of epithelial cells, typically goblet cells, that overproduce mucin, the main component of mucus. Normally, mucins help with the function of healthy cells, but in mucinous carcinomas, the mucins that surround the cancer cells become part of the tumor.

There are two types of primary mucinous tumors that grow in alveolar spaces: mucinous adenocarcinoma, previously known as BAC, and colloid adenocarcinoma. Compared to other lung cancer subtypes, mucinous adenocarcinoma differs in its secretion mechanisms, which cause mucus overproduction. This may be due to gene expressions that dysregulate signaling pathways. They typically present Kirsten rat sarcoma viral oncogene homolog (KRAS) mutations and commonly lack thyroid transcription factor‐1 expression (TTF1) [7]. Mucinous adenocarcinoma of the lung is morphologically characterized by tall columnar cells with abundant cytoplasm that contain varying amounts of mucin [8].

According to the recent classification, mucinous adenocarcinoma has three different types: invasive mucinous adenocarcinoma, when the tumor is more than 3 cm in size and/or shows an acinar invasive pattern larger than 0.5 cm; invasive mucinous adenocarcinoma, when the mass measures less than 3 cm and does not show invasion; and minimally invasive adenocarcinoma if there is an invasive component less than 0.5 cm [9].

Pulmonary colloid adenocarcinoma is another type of lung cancer characterized by an abundant amount of mucin production that distends into the alveolar spaces and destroys their walls. Colloid carcinomas are generally positive for CK7 and CDX2 and weak or negative for TTF1, napsin A and EMA (MUC1) [10]. These tumors are typically indolent and have a favorable prognosis after surgical resection [11].

Both mucinous adenocarcinoma and colloid carcinoma cause an overproduction of mucin, which results in mucin‐filled spaces within the tumor, which in turn forms a barrier that blocks the penetration of chemotherapeutic drugs into the tumor cells [12]. High mucin content can also alter the tumor microenvironment, potentially inducing hypoxia, pH fluctuations, and alterations in the extracellular matrix [12].

Differentiating colloid adenocarcinoma from invasive mucinous adenocarcinoma is challenging, given their similar morphological characteristics. However, they have distinct features: mucinous adenocarcinoma histologically presents with goblet‐shaped or columnar cells that adhere to the walls. In contrast, in pulmonary colloid adenocarcinoma, cells produce mucin which forms pools that distend and destroy the underlying alveolar architecture. Recently, there has been a push to reclassify mucinous adenocarcinoma as low‐grade mucinous tumors, which may help clarify and offer more specific classifications to these tumors [6].

Lesions with these histologic and immunophenotypic characteristics usually behave in an indolent fashion and are best treated by complete surgical resection. One study compared the treatment and prognosis of early‐stage pulmonary mucinous adenocarcinoma (Stage 1) when treated with surgical methods and adjuvant chemotherapy [13]. The results showed that compared to surgery alone, adjuvant chemotherapy had a negative impact on overall survival and cancer‐specific survival. Our patient has so far had a good prognosis, showing that surgical resection may be the best treatment option.

Like our patient, most cases of pulmonary colloid adenocarcinoma are asymptomatic. A case report of a 79‐year‐old man whose chest CT revealed an enlarging, well‐defined nodule in his right upper lobe (RUL) underwent a RUL wedge resection [14]. Immunohistochemistry revealed that the tumor cells were positive for CEA, CK7, and TTF‐1 and negative for CK20, confirming pulmonary colloid adenocarcinoma. He was discharged and has had no evidence of recurrence.

There have also been cases of mucinous adenocarcinoma with poor prognosis. For instance, a 60‐year‐old Chinese woman presented with a fever, cough, and yellow sputum [15]. A chest CT revealed a lesion in the right lung, and a fiberoptic bronchoscope examination revealed a mucinous membrane with black spots in the RUL anterior segmental bronchus. As the respiratory symptoms became increasingly severe, the patient underwent a right pneumonectomy. Postoperative pathological analysis revealed lung mucinous adenocarcinoma. Adjuvant chemotherapy included four cycles of Taxol and cisplatin. However, she eventually succumbed to the disease.

Our understanding of low‐grade mucinous tumors is gleaned from small case reports and series. Because of their asymptomatic presentation, diagnosis and treatment can be difficult. Further research and case reports will help improve their diagnosis and treatment. New areas of research that aim at developing drugs that target specific biomarkers and genes will help create more personalized treatments.

## Consent

All the patients allowed personal data processing, and informed consent was obtained from all individual participants included in the study.

## Conflicts of Interest

The authors declare no conflicts of interest.

## Funding

No funding was received for this manuscript

## Data Availability

The data that support the findings of this study are available on request from the corresponding author. The data are not publicly available due to privacy or ethical restrictions.
